# Eco-Friendly Natural Rubber–Jute Composites for the Footwear Industry

**DOI:** 10.3390/polym15204183

**Published:** 2023-10-21

**Authors:** Giovanni Barrera Torres, Carlos T. Hiranobe, Erivaldo Antonio da Silva, Guilherme P. Cardim, Henrique P. Cardim, Flavio C. Cabrera, Elizabeth R. Lozada, Carlos M. Gutierrez-Aguilar, Juan C. Sánchez, Jaime A. Jaramillo Carvalho, Aldo E. Job, Renivaldo J. Santos

**Affiliations:** 1 Industrial Design Engineering Department, Faculty of Arts and Humanities, Metropolitan Institute of Technology (ITM), Medellín 050036, Colombia; giovannibarrera@itm.edu.co (G.B.T.); carlosgutierrez@itm.edu.co (C.M.G.-A.); 2 School of Engineering and Sciences, São Paulo State University (UNESP), Rosana 19274-000, SP, Brazil; carlos.hiranobe@unesp.br (C.T.H.); guilherme.cardim@unesp.br (G.P.C.); fc.cabrera@unesp.br (F.C.C.); 3 Department of Cartography, School of Science and Technology, São Paulo State University (UNESP), Presidente Prudente 19060-900, SP, Brazil; erivaldo.silva@unesp.br; 4 Postgraduate Program in Science and Technology of Materials (POSMAT), School of Engineering and Sciences, São Paulo State University (UNESP), Rosana 19274-000, SP, Brazil; henrique.cardim@unesp.br; 5 Postgraduate Program in Sustainable Development, Faculty of Exact and Applied Sciences, Metropolitan Institute of Technology (ITM), Medellín 050036, Colombia; eliza.rico.lozada@gmail.com; 6 Advanced Manufacturing Technology Center (SENA), Medellín 050036, Colombia; jcsanchezg@sena.edu.co (J.C.S.); jjaramilloc@sena.edu.co (J.A.J.C.); 7 Mechanical Department, Pascual Bravo University Institution (IUPB), Medellín 050036, Colombia; 8 Department of Physics, School of Science and Technology, São Paulo State University (UNESP), Presidente Prudente 19060-900, SP, Brazil; aldo.job@unesp.br

**Keywords:** natural rubber, rubber composites, jute fibers, eco-friendly composites

## Abstract

Nowadays, biocomposites represent a new generation of materials that are environmentally friendly, cost-effective, low-density, and not derived from petroleum. They have been widely used to protect the environment and generate new alternatives in the polymer industry. In this study, we incorporated untreated jute fibers (UJFs) and alkaline-treated jute fibers (TJFs) at 1–5 and 10 phr into TSR 10 natural rubber as reinforcement fillers. These composites were produced to be used in countersole shoes manufacturing. Untreated fibers were compared to those treated with 10% sodium hydroxide. The alkali treatment allowed the incorporation of fibers without compromising their mechanical properties. The TJF samples exhibited 8% less hardness, 70% more tensile strength, and the same flexibility compared to their pure rubber counterparts. Thanks to their properties and ergonomic appearance, the composites obtained here can be useful in many applications: construction materials (sound insulating boards, and flooring materials), the automotive industry (interior moldings), the footwear industry (shoe soles), and anti-static moldings. These new compounds can be employed in innovative processes to reduce their carbon footprint and negative impact on our planet. Using the Lorenz–Park equation, the loaded composites examined in this study exhibited values above 0.7, which means a competitive load–rubber interaction. Scanning electron microscopy (SEM) was used to investigate the morphology of the composites in detail.

## 1. Introduction

The overexploitation of oil resources—which are estimated to last no more than 60 years at the current consumption rate [1]—and the depletion of non-renewable resources have encouraged humans to reduce their environmental impacts. This has produced growing ecological awareness in the fields of healthcare, sustainability, and environmental conservation. This has also led to the prioritization of biodegradable and sustainable raw materials and the development of much more environmentally friendly alternatives [2,3,4,5] to replace conventional industrial applications that have low sustainability. Raw materials like natural rubber and plant fibers are considered suitable replacements for synthetic compounds derived from fossil sources that are currently used in the production of many goods. The global interest in natural fibers for various applications has grown significantly in the past decade. In 2018, the global production of natural fibers reached 32 million metric tons [6]. The main reason behind the increased supply and demand for these raw materials in the market is the fact that their physical and mechanical properties make them suitable for a wide range of industrial purposes [7]. 

Compared to their inorganic counterparts, natural fibers are abundant renewable resources. They are readily biodegradable, nontoxic, nonabrasive, low-density, low-cost [8,9,10,11,12,13,14], not harmful to human health [15,16], and resistant to corrosion [17], alkalinity, and most organic acids. However, they can be destroyed by solid mineral acids [18]. Natural vegetable fibers (VFs) have been widely used to reinforce polymeric composite materials. Moreover, VFs (in percentages previously analyzed in several studies) are preferred over inorganic fibers in industrial applications. New materials can incorporate natural fibers to make the most of their low cost; versatility; abundance; environmental attributes (i.e., renewable, recyclable, and biodegradable) [19,20]; mechanical properties (e.g., high tensile strength, flexural modulus, and low density); high electrical resistance; thermal and acoustic insulation; and fracture resistance [21]. Although natural fibers offer multiple benefits, studies show that their mechanical properties are worse than those of synthetic fibers, and their hydrophilic nature makes them less compatible with polymeric materials. Therefore, they require a surface treatment to improve their adhesion and coupling to the polymeric phase.

At present, more environmentally friendly composites with natural rubber are being developed. The global production of polymers from sustainable natural sources grew more than 400% between 2016 and 2019 [22,23]. These materials—also known as green composites, eco-composites, bio-composites, or eco-friendly composites—have ecological and environmental properties comparable to those of their conventional counterparts [24,25,26]. Natural rubber is an attractive and environmentally friendly option for developing eco-friendly composites. After all, rubber composites reinforced with vegetable fibers are currently employed in several industries due to their engineering properties, low cost, and environmental characteristics [27]. They also reduce the use of dangerous active fillers [28]. Natural rubber composites reinforced with natural fibers offer significant mechanical improvements: higher strength and modulus, decreased elongation at break, improved creep resistance over particulate-filled rubber, increased hardness, and substantially better cut-and-tear resistance [29,30].

Composite materials made of natural rubber and short fibers have been used in multiple industries to develop several commercially viable products, such as conveyor belts, soles, and V-belts [31,32]—optimizing their strength, stiffness, elastic modulus, and damping [33]. The most important properties of these fibers are strength, dispersion, aspect ratio, content, orientation, length [34], rubber/fiber interaction (i.e., the compatibility of these phases), and interfacial adhesion. The interfacial interaction between these fibers and a matrix is critical in producing high-performance composites and their applications [35,36]. Weak interfacial adhesion means incompatibility between the composite phases, leading to poor mechanical properties [37].

The most common chemical process applied to natural fibers is an alkali treatment [19] that uses sodium hydroxide (NaOH) [38]. The fibers are immersed in NaOH solutions at specific concentrations to remove lignin and surface impurities (such as pectins and oils), which act as barriers and reduce the hydrophilic behavior of the hydroxyl groups in the fibers. As a result, the roughness of the fiber surface is increased [39]. Fibers pretreated with alkaline agents, even in low proportions, have been shown to favor the mechanical properties of composites, providing higher strength, stiffness, and a better interfacial ratio thanks to a greater exposure of the cellulose and higher surface energy, which produce better wettability and compatibility [40].

These composite materials have been implemented, for example, in footwear soles as reinforcement, improving their coefficient of friction (COF). However, said COF decreases when the soles absorb water, while the other mechanical properties are preserved due to the fiber treatments mentioned above. Usually, the shoe sole industry has used ethylene-vinyl acetate (EVA) because of its properties, density, and elastic modulus, which are relatively good indicators of its shock-absorption efficiency. Structures made of EVA foams can improve the shock-absorption properties of footwear, thus reducing the impact force and improving its durability (stiffness) [41].

This paper describes the mechanical and physical properties of natural rubber filled with low proportions of jute fibers (from 1 to 5 phr and also 10 phr), with and without an alkali treatment. These composites were prepared based on previous studies published over the last eleven years. However, said studies have explored composites with more than 5 phr of natural fiber in the mixture—not lower proportions. Therefore, this study investigates lower filler proportions to establish how they can affect the mechanical behavior of a composite that can be used in the shoe industry.

In this study, we evaluated some conditions to improve the mechanical response of composites made of NR by incorporating micronized jute fibers smaller than 110 µm into the matrices. We examined the degree of reinforcement thus produced by calculating the crosslinking densities of the composites using the Flory–Rehner method and the Lorenz–Park equation (to determine the interfacial interaction between the fillers and the polymeric matrix). These densities were compared to those obtained by the Mooney–Rivlin method, as shown below. This approach enables a better understanding of the matrix–filler interaction.

## 2. Materials and Methods

In Ecuador, jute fibers are used in the textile industry because they have two characteristics that make them very attractive for industrial applications: excellent quality and low cost. This material has been processed as conventional tissue, i.e., dried at room temperature before treatments and mixture. This study used Technical Specified Rubber (TSR) 10, with a Mooney viscosity between 70 and 90. Two reagents were used in the vulcanization process (i.e., stearic acid and zinc oxide); sulfur was the curing system; and N-tert-butyl-2-benzothiazolime sulfonamide (TTBS) was the accelerator.

The composites were prepared as follows. First, the fibers were acquired, cut, and electromechanically sieved (with a 110 ASTM sieve). Then, the natural rubber was mixed with two kinds of jute fibers: untreated (UJFs) and treated with an alkali (TJFs). The vulcanizing agents were subsequently added to the mixture. When the jute fibers had been mixed and dispersed in the NR, the vulcanization activators were added. Afterward, the composite was mixed in a two-roll rubber mill for 20 min at 60 °C and a friction ratio of 1.0:1.25. The fibers were added in proportions from 1 to 5 phr and then 10 phr (parts per hundred rubber). These proportions of material were selected considering the lowest percentages of jute fibers that have been employed in other studies that have examined their response to competitive stress. Therefore, this study applied different experimental treatments to optimize the response of the composite using the lowest possible amount of jute fibers. The composites were formed in a thermal press using a rectangular plate (dimensions: 5.90 × 4.33 × 0.078 inches) under 430 psi of pressure and a temperature of 150 °C. The optimum vulcanization time (t90) was established using an oscillating disc rheometer according to ASTM D2084 [42] and ASTM D5289 [43]. The composites thus obtained were labelled NR/TJF (i.e., natural rubber with treated jute fibers) and NR/UJF (i.e., natural rubber with untreated jute fibers).

The number of reagents to be incorporated into the rubber mixture was established based on parameters that had been previously established (see Table 1). In the first stage, zinc oxide and stearic acid were incorporated into NR until it was completely homogenized. The mixture was left to stand for 24 h to enable the formation of zinc stearate and facilitate the action of the accelerators and sulfur. In the second stage, after the treated and untreated fibers were added, paraffinic oil was incorporated to improve the dispersion of the sulfur and the accelerator (N-tert-butyl-2-benzothiazolime sulfonamide, TTBS).

To understand the vulcanization behavior, rheometry experiments were performed using a rheometer (produced by Team Equipment Ltda in Sao Pablo, Brazil) with an isotherm system at 150 °C and a disc oscillation of 1°. The curing parameters were determined based on the curves obtained according to ASTM D 2084. Abrasion tests were conducted in triplicate using a rubber abrasion tester (with a revolving drum) according to ASTM D 5963 [44]. The samples were subjected to a friction distance of 40 m, which corresponds to 84 drum rotations under a force of 5 ± 0.2 N (1.125 ± 0.02 lbf). Hardness tests were performed using a Kiltler durometer, (Sao Pablo, Brazil) with Shore A and Shore OO scales according to ASTM D 2240 [45].

Stress–strain tests were performed on Die C dumbbell test specimens using a Lloyd AGS-X 20 KN universal testing machine (Shimazu, Kyoto, Japan) according to ASTM D412, Method A, at 500 mm/min, with a load of 0.5 kN and an internal deformation transducer [46]. The morphology on the surface of the composites and jute fibers was analyzed using a Carls Zeiss EVO LS15 scanning electron microscope (Oberkochen, Germany) with back-scattering detector, high-vacuum mode, and a potential of 20 kV. A Quorum Q150R ES sputter coater (Lewes, UK) was employed to previously cover the samples with a thin gold film. A Bareiss Shore A and Shore OO durometer (Oberdischingen, Germany) was used following ASTM D2240 to determine the hardness of the samples.

The swelling method was adopted to determine the crosslinking density of the composites. The samples, with a mass of approximately 0.25 ± 0.05 g each, were weighed and then immersed in toluene for five days. After this period, the specimens were removed, surface-dried with absorbent paper, and weighed again. They were then placed in an oven at 60 °C for 24 h and weighed one more time. The crosslinking density was calculated according to Equation (1), which was developed by Flory and Rehner [47,48]:

(1) $$\eta =\frac{-(\ln\left(1-V_{b}\right)+V_{b}+X(V_{b}{)}^{2})}{\left(p_{b})(V_{0})(V_{b}^{\frac{1}{3}}-\frac{V_{b}}{2}\right)},$$

where *V_b_* is the volume fraction of the polymer in the swollen gel at equilibrium; *X*, the polymer–solvent interaction parameter; *p_b_*, the density of the polymer; and *V*_0_, the molar volume of the solvent.

### Alkaline Treatment

The two composites—natural rubber with untreated jute fibers (i.e., NR/UJF) and natural rubber with treated jute fibers (i.e., NR/TJF)—were produced by mixing NR and jute fibers at different proportions, ranging from 1 to 5 and also at 10 phr. The jute fibers were dried for 24 h at 80 °C in an oven and then micronized to reduce their particle size. The alkaline treatment, following the procedure conducted by de Paiva et al. [40], was carried out in 10 wt.% of sodium hydroxide (NaOH) with 1.0 g of fiber totally immersed in 10 mL of hydroxide solution for 24 h at 25 °C and neutralized for one hour with 2%v of acetic acid until a pH of 7.0 was obtained, thus promoting delignification. Then, the jute fibers were dried in an oven for 48 h at 80 °C. Afterward, they underwent an electromechanical sieving process using an 110 ASTM (Caliche G corp. Medellin, Colombia) sieve so that they were ready to be mixed with natural rubber in the two-roll mill.

## 3. Results and Discussion

### 3.1. Evaluation of the Fibers

**Figure 1:**
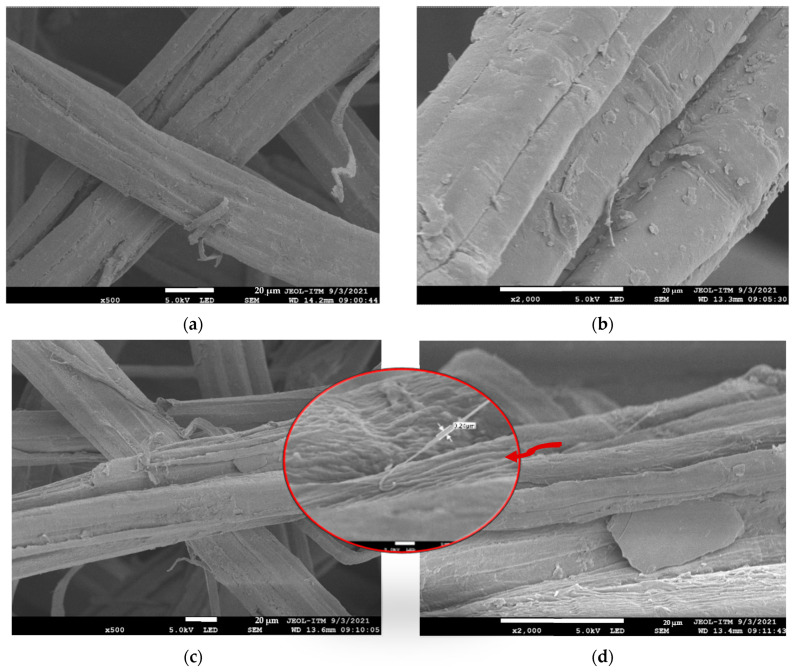
SEM micrographs of UJFs at 500× (**a**) and 2000× (**b**) magnifications and TJFs at 500× (**c**) and 2000× (**d**) magnifications.

In some applications—mainly rubber products—filler particles such as carbon black and SiO_2_ are incorporated to improve mechanical properties. Nowadays, natural fibers have become a viable alternative for this purpose. Figure 1 presents four SEM micrographs of the surface of the jute fibers employed here. The UJFs (Figure 1a,b) show a slightly rough surface, which is generally associated with the constituent pectin, wax, and oil wax [49]. These surface impurities limit their adhesion to the matrix, weakening the mechanical properties of the composites. In contrast, the surfaces of the TJFs (Figure 1c,d) were cleaned efficiently and are free from impurities, thus improving their surface topography, increasing the contact area, enabling a better interaction between the filler and the rubber matrix in treated composites, and achieving better mechanical resistance than their untreated counterparts.

Furthermore, the rugosity of the TJFs was increased, possibly improving the efficient surface area for contact with the rubber matrix. Hence, there is a slight improvement in mechanical properties.

### 3.2. Rheometry

Table 2 details the parameter values measured in the composites during the rheometric tests: maximum and minimum torque, torque variation, and optimum cure time (t90). This table shows that the treated jute fibers presented a reduction in minimum (ML) and maximum (MH) torque, as well as the lowest t90 values among the samples examined here.

This reduction in torque is directly related to the lower viscosity produced by adding the filler. A more homogeneous response was obtained in the treated jute fibers compared to the pure rubber (no filler) and the samples with untreated fibers. A similar result was reported by Santos et al. [10] using SCBA samples without silane agent coupling. In that case, the viscosity was increased due to the reinforcing nature of the filler, and an increase in maximum torque and t90 was directly related to the degree of curing, i.e., the number of cross-links formed during the vulcanization process. This means greater stiffness, which may be due to both the presence of the loading and the formation of a higher number of (chemical and/or physical) cross-links [50].

The relation between maximum and minimum torque (ΔM) defines the degree of reinforcement caused by the incorporation of the filler, which was enhanced thanks to the alkaline treatment. Likewise, the lower ML and t90 values in the optimized (treated) samples indicate that the alkaline process favors an earlier vulcanization of the composites. Still, the higher t90 values of the composites with fillers result from the increased viscosity and interaction between the jute fibers and natural rubber homogenized by mastication.

The scorch time (t2) and optimum cure time (t90) were markedly lower in the NR/TJF composites due to a better interaction between the matrix and the treated natural fibers (compared to their untreated counterparts). Conversely, to prepare the NR/UJF samples, the composites should remain for a longer period in the two-roll mill. Thus, the decreasing trend in t2 and t90 in the NR/TJF composites was closely related to the generation of a more significant amount of heat due to additional friction. The NR/TJF composites exhibited shorter t2 and t90 than their NR/UJF counterparts, which is attributed to an improved dispersion of fibers in the NR matrix of the former. Therefore, the incorporation of the treated jute fibers can increase crosslinking and improve the mechanical properties of the composites as discussed below.

### 3.3. Determining Crosslinking Density

**Figure 2:**
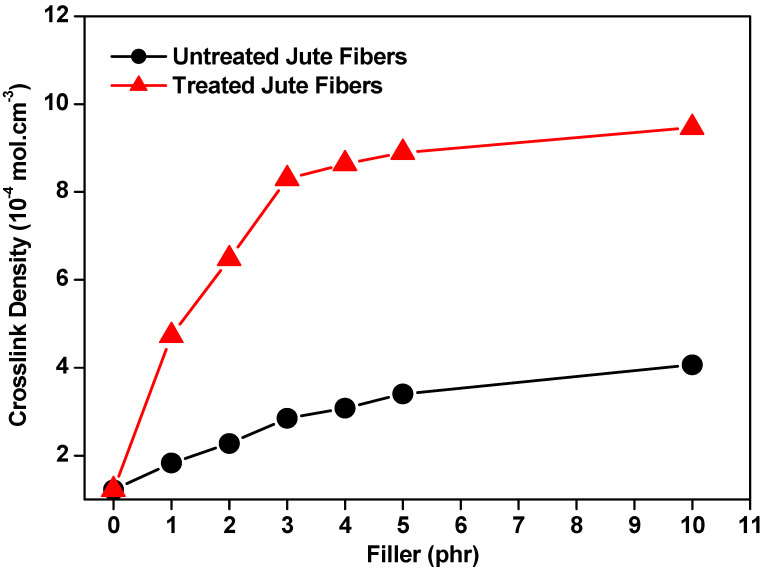
Crosslinking density of NR/UJF and NR/TJF composites.

Figure 2 shows the crosslinking density obtained in the vulcanized composites with treated and untreated jute fibers. A marked increase in said density can be observed in the composites that contain treated fibers (i.e., NR/TJF). These results can be attributed to a possible reaction of better adhesion caused by the additives during the vulcanization process, increasing the cure rate and, consequently, reducing the time needed to produce a higher density of cross-links.

The untreated fibers caused a reduction in crosslinking density, possibly due to their more limited interaction with the natural rubber. In contrast, the treated fibers presented a linear growth in crosslinking density as the proportion of filler was increased up to 4 phr.

### 3.4. Analysis of Rubber/Jute Fiber Interactions Using the Lorenz–Park Equation

The interaction between the jute fibers and the rubber matrix was determined employing the method developed by Lorenz and Park [51] and the parameters obtained from the solvent swelling tests used in Equation (2) [52]:

(2) $$\frac{Q_{l}}{Q_{r}}={ae}^{-z}+b,$$

where:

- Q = weight of toluene absorbed per gram of rubber, where subscripts l and r denote the composites vulcanized with a loading and pure rubber, respectively;
- z = ratio of filling mass per unit mass of rubber;
- a and b = constants.

The value of Q was calculated using Equation (3):

(3) $$Q=\frac{w_{s}-w_{d}}{w_{r}\times 100/w_{F}},$$

where:

- W_s_ = weight of the swollen composite when the balance between the organic solvent and the polymer is achieved;
- W_d_ = weight of the dry composite;
- W_r_ = weight of the rubber in the dry composite;
- W_F_ = total weight of the formulation.

### 3.5. Analysis of Rubber/Loading Interactions Using the Lorentz–Park Equation

The swelling data of the composites in an organic solvent (Flory–Rehner) were used in the calculation of the Qf/Qg ratio applying Equation (2). The Qf/Qg ratio reflects the restriction to swelling by the rubber matrix in the vicinity of the loaded particles. When rubber is immersed in the organic solvent, the latter tends to penetrate and degrade the regions that are not firmly bound to the loadings or cross-linked. Although rubber expands in response to the solvent, firmly adhered loaded particles can limit this expansion. The higher the Qf/Qg value, the weaker the load/matrix interaction because the latter tends to be load/load. And z is the ratio between the amount of loading and the amount of rubber (phr). Hence, an increase in the amount of loading in the composite generates an exponential reduction in z [53,54].

**Figure 3:**
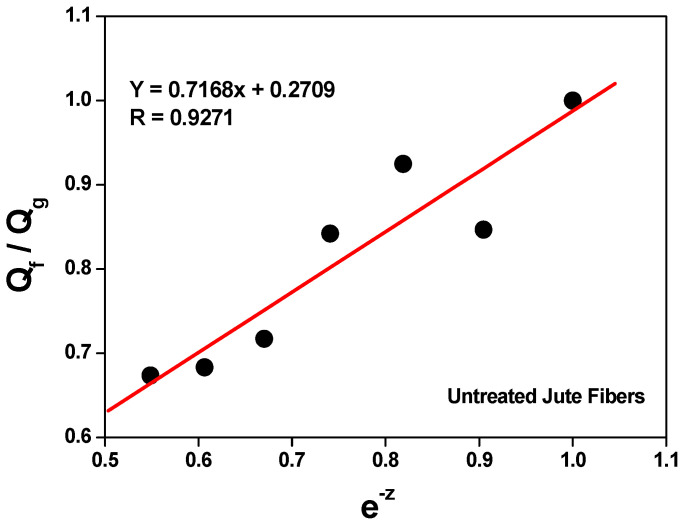
Variation in Qf/Qg versus e^−z^ in composites vulcanized with untreated jute fiber.

**Figure 4:**
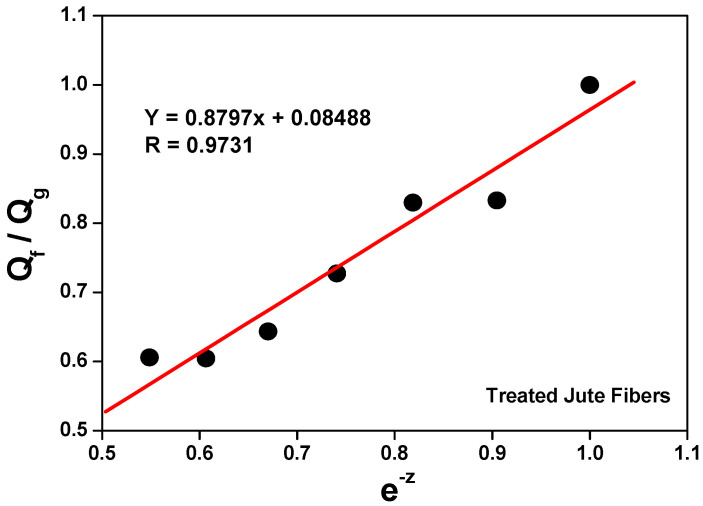
Variation in Qf/Qg versus e^−z^ in composites vulcanized with treated jute fibers.

Figure 3 and Figure 4 show a linear relationship between the curve of Qf/Qg and that of e-z. As more loading is added, the Qf/Qg ratio increases as a function of e^−z^. This behavior is due to the fact that Qf values are related to the swelling of the loaded rubber. Therefore, the loading works as an obstruction to solvent penetration due to a good matrix/loading interaction. Parameters a and b equal 0.7168 and 0.2709, respectively, with a correlation coefficient (R) of 0.9271 for untreated jute fibers, and a correlation coefficient (R) of 0.9731 (Y = 0.8797x + 0.08488) for treated jute fibers. According to Lorenz and Park, since the value of parameter a is greater than 0.7 and the slope of a is higher, the rubber/loading interaction is strong. The value of said parameter (much higher than 0.7) denotes a firm rubber/fiber interaction.

**Figure 5:**
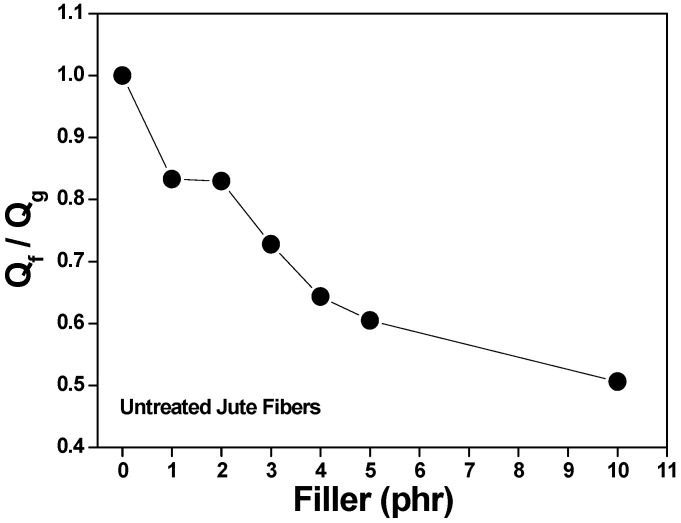
Effect of filler loading on the Qf/Qg of composites vulcanized with untreated jute fibers.

**Figure 6:**
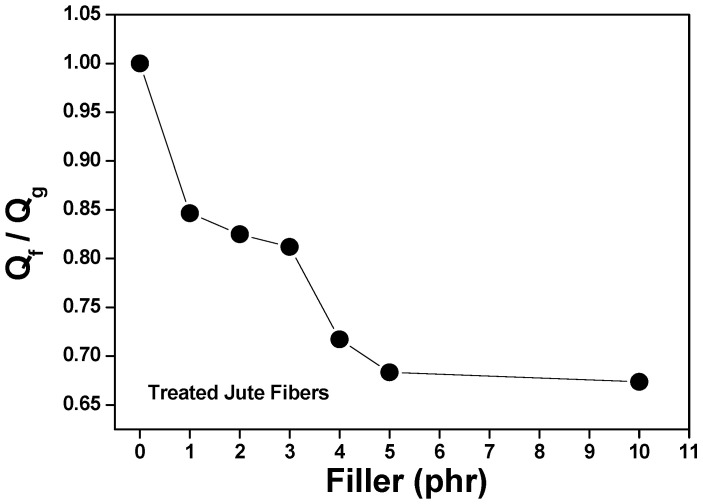
Effect of filler loading on the Qf/Qg of composites vulcanized with treated jute fibers.

Although all the composites have the same formulation and crosslinking system, Figure 5 and Figure 6 show a decrease in the Qf/Qg ratio as the amount of loading was increased; in other words, less solvent penetrated the more loaded composites. This is associated with improved mechanical properties and attributed to a strong interfacial interaction between the elastomeric chains and the fibers, which was generated by a good dispersion of said fibers in the matrix.

### 3.6. Shore OO Hardness

Hardness tests were conducted to evaluate the relative surface resistance of the compounds. Hardness was determined on the Shore OO scale, which suggests that the specimens presented a low density similar to 40 Shore A hardness. This is lower than the results obtained in the pure material and in mixtures of natural rubber with grass fibers and ashes [55,56], in which hardness values above 40 Shore A were produced with fiber loadings higher than 10 phr [57]. Materials with Shore OO hardness are usually employed in footwear and cushioning against impact in school floors and gel insoles. Table 3 reports the Shore OO values obtained in this study. It was possible to determine that a low percentage of fibers in the samples influenced the hardness of the latter, which was corroborated in an analysis of relative density. Consequently, the compounds obtained here could replace synthetic materials in the footwear industry.

These results allow us to evaluate the hardness of the composites as a function of the percentage of jute fibers in them. We can observe a similar behavior in the values of the composites with jute fibers without surface treatment (NR/UJF) and those with treated fibers (NR/TJF), which suggests that the addition of jute fibers did not increase the stiffness of the composites as indicated in the literature [2,3].

Interestingly, the composites exhibited a slight reduction in hardness compared to the unreinforced natural rubber blend (80.8 Shore OO). This can be attributed to a more significant interaction between the fiber and the matrix, occupying the volume of the matrix but with lower density, which results in a softer material [58]—except for NR/UJF 4 phr, which showed an increase in hardness up to 81.1 Shore OO, the highest hardness value among all the composites analyzed here.

### 3.7. Abrasion Resistance

The abrasion resistance tests were performed according to the UNE-ISO 4649:2013 standard [5]. These tests used a Maqtest abrasion tester, at a constant rotation of 40 rpm. This equipment belongs to the LACPA Laboratory of the Design and Advanced Manufacturing Center at SENA (Itagüí, Antioquia, Colombia). Its drum has a diameter of 150 mm and an abrasion path of 40 mm. The force applied to the body of the specimen was 5.0 N, with a dip angle of 3° with respect to the vertical axis along the center of the drum. The tests were performed in triplicate. Equation (4) was used to determine the abrasion resistance index:

(4) $$IR=\frac{m_{1}d_{t}}{m_{t}d_{1}}\;x100,$$

where:

- Iar = abrasion resistance index (%);
- *m*_1_ = initial mass of reference rubber (mg);
- *m_t_* = initial mass of test rubber (mg);
- *d*_1_ = initial mass of reference rubber (mg/cm^3^);
- *d_t_* = final mass of test rubber (mg/cm^3^).

**Figure 7:**
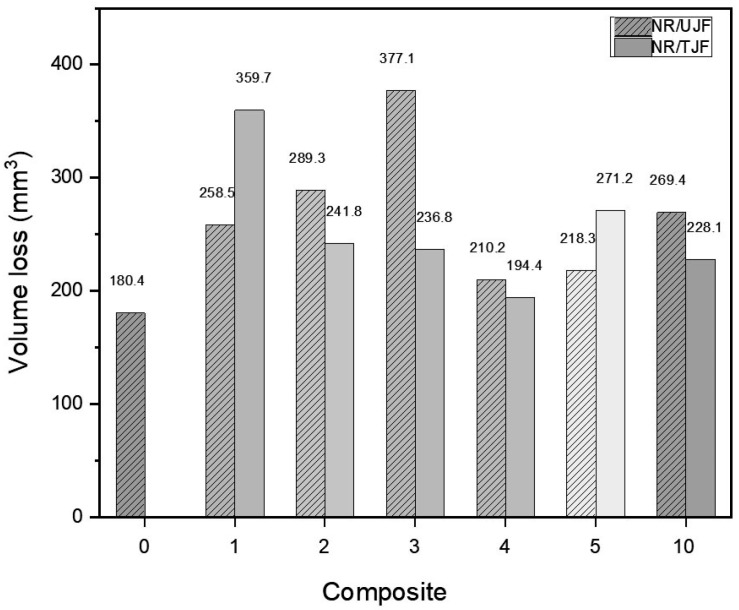
Volume lost from the NR/UJF and NR/TJF composites during the abrasion tests.

Table 4 reports the results of the abrasion tests performed on the composites made of natural rubber combined with untreated (NR/UJF) and treated (NR/TJF) jute fibers. To evaluate the results qualitatively, Figure 7 shows the amount of material lost from the samples due to abrasion.

The results show that incorporating jute fibers decreases the abrasion resistance of all the samples, although it is generally lower in the specimens containing UJF reinforcement. This mass loss is associated with stress points due to the interaction between the fibers added as reinforcement [10,59]. The samples with the lowest abrasion resistance were NR/UJF 3 phr (with a volume loss of 377.1 mm^3^) and NR/TJF 1 phr (with a volume loss of 359.7 mm^3^). The specimens that contained 4 phr showed the lowest mass loss, i.e., higher abrasion resistance compared to specimens with other amounts of fiber both with and without treatment.

**Figure 8:**
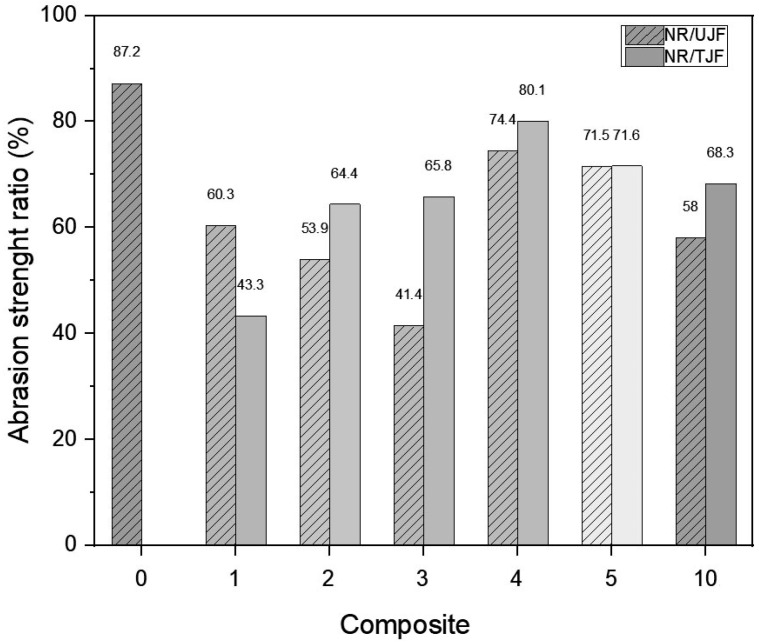
Abrasion resistance indices of the composites.

Figure 8 shows the abrasion resistance indices of the specimens as a function of the amount of reinforcement incorporated into each rubber mixture. The mixtures with 4 and 5 phr exhibited the highest resistance values (above 70%) among all the compounds. The NR/TJF composites with 4 and 5 phr presented lower abrasion resistance indices (8% and 18% lower, respectively) than those of the natural rubber mixture without reinforcement. The values obtained for the NR/TJF samples show better frictional resistance properties compared to their counterparts with untreated filler. This can be attributed to a good dispersion of the reinforcing material, as well as a better adhesion between the treated fibers and the elastomeric matrix, thus decreasing the space between particles [13,60,61].

### 3.8. Tensile Strength

**Figure 9:**
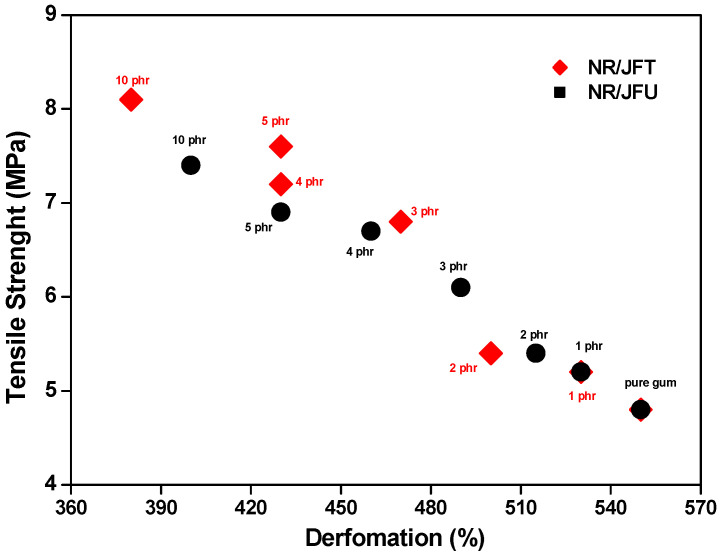
Tensile strength of composites.

Figure 9 details the tensile and deformation values measured in the NR/UJF and NR/TJF composites. The loaded specimens exhibited worse tensile/strain properties than the natural rubber mixture without jute fibers, which can be attributed to the incompatibility between the hydrophilic jute fibers and the hydrophobic matrix, as reported in [2,12]. Importantly, the NR/TJF composite with 10 phr filler exhibited tensile/strain properties (i.e., 8.1 MPa and 380%, respectively) that are similar to those of the natural rubber blend without aggregates (i.e., 4.8 MPa and 550%, respectively). This fact is associated with the removal of impurities and hydrophilic components (hemicellulose, lignin, pectins, and waxes) caused by the alkalinization treatment previously performed on the fibers. In general, said treatment reduces the diameter of the fibers, increasing their aspect ratio as well as their effective surface area, thus improving interfacial adhesion, which restricts matrix movement and transfers stress to the reinforcement phase. This can also be the result of a more adequate fiber dispersion compared to its counterparts [13,14,15].

### 3.9. Morphology of the Composites and Jute Fibers

**Figure 10:**
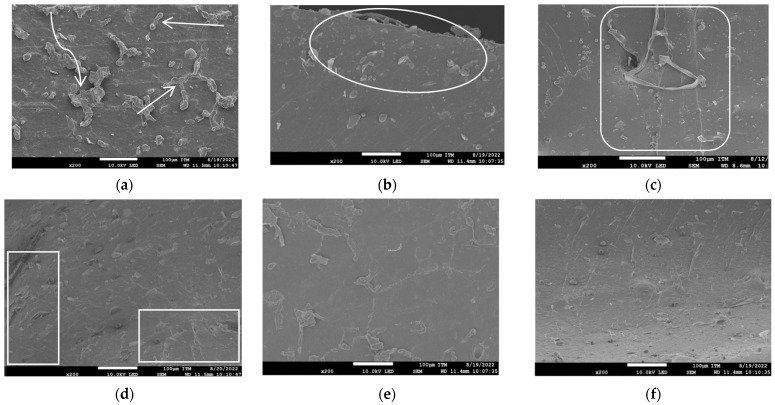
SEM micrographs with 200 magnification of NR/UJF at 1 phr (**a**), 5 phr (**b**), and 10 phr (**c**), and NR/TJF at 1 phr (**d**), 5 phr (**e**), and 10 phr (**f**).

Scanning Electron Microscopy (SEM) was employed to analyze the surface morphologies of the samples, as shown in Figure 10. Evidently, in the untreated jute fiber samples (Figure 10a–c), the fibers are visibly detached from the NR matrix. Indeed, exposed fibers can be observed in Figure 10a–c. In the cross-section in Figure 10b, there are visible fibers on the surface and throughout the body of the sample, potentially due to weak surface adhesion and a potential reduction in crosslinking. This could result in lower mechanical strength.

Conversely, the cross-sections of the NR/TJF samples (Figure 10d–f) exhibit improved adhesion between the fibers and rubber. There is a noticeable decrease in fiber exposure, which can be attributed to an enhanced interaction at the fiber–rubber interface. This leads to better tensile and mechanical deformation responses in the final outcome.

## 4. Conclusions

The proportions of jute added to NR in this study were low compared to those in many publications where other fibers have been blended in proportions above 30 phr. Nevertheless, the composites prepared in this study showed tensile and deformation properties that can be exploited in sustainable and economic applications for the industrial sector, specifically footwear manufacturing.

These low concentrations of jute fibers did not result in significantly different physical–mechanical properties (stress–strain). However, in low proportions, they were fundamental to obtaining hardness values lower than Shore A, which indicates a reduction in relative density. As a consequence, the composites exhibited Shore OO hardness values similar to those of EVA, which is used in the footwear industry to make soles and protective elements for children’s playgrounds.

This study opens up a path to better understanding the influence of low proportions of natural fibers on polymeric composites for sustainable industrial applications.

## Figures and Tables

**Table 1:** Formulations of NR composites with different proportions of jute fibers.

Function	NR/TJF ^1^	NR/UJF ^2^	Phr ^3^
Matrix	NR	NR	100
Activator	Stearic acid	Stearic acid	4.0
Activator	ZnO	ZnO	6.0
(Filler) jute fibers	TJF ^1^	UJF ^2^	0, 1, 2, 3, 4, 5, 10
Curing agent	S	S	1.75
Lubricant	Paraffin	Paraffin	3.5
Accelerator	TTBS ^4^	TTBS	0.8

^1^ Treated jute fibers, ^2^ untreated jute fibers, ^3^ per hundred rubber, ^4^ N-tert-butyl-2-benzothiazolime sulfonamide.

**Table 2:** Rheometric parameters of the NR/UJF and NR/TJF composites.

Composite	M_L_ (dNm)	M_H_ (dNm)	∆M (dNm)	t_90_ (dNm)
Pure rubber	1.96 ± 0.16	4.33 ± 2.16	3.04 ± 2.06	6.91± 0.06
NR/UJF 1.0	1.26 ± 0.12	3.49 ± 1.56	2.23 ± 0.06	8.92 ± 0.11
NR/UJF 2.0	1.28 ± 0.06	3.52 ± 2.26	2.24 ± 2.25	10.29 ± 0.11
NR/UJF 3.0	1.45 ± 0.13	3.47 ± 2.16	2.02 ± 0.06	10.36 ± 0.02
NR/UJF 4.0	1.49 ± 0.12	3.46 ± 1.26	1.97 ± 1.14	11.04 ± 0.10
NR/UJF 5.0	1.39 ± 0.12	3.49 ± 2.16	2.10 ± 2.03	10.42 ± 0.12
NR/UJF 10	1.23 ± 0.10	3.26 ± 2.06	2.03 ± 2.01	10.75 ± 0.08
NR/TJF 1.0	0.85 ± 0.13	1.81 ± 1.36	0.96 ± 1.03	8.26 ± 0.02
NR/TJF 2.0	0.86 ± 0.09	2.24 ± 2.28	1.38 ± 2.16	9.22 ± 0.05
NR/TJF 3.0	0.98 ± 0.14	3.05 ± 2.07	2.07 ± 2.06	8.95 ± 0.06
NR/TJF 4.0	1.09 ± 0.14	3.06 ± 1.46	1.97 ± 1.33	9.82 ± 0.09
NR/TJF 5.0	1.10 ± 0.11	3.61 ± 2.37	2.51 ± 2.25	7.35 ± 0.08
NR/TJF 10	0.93 ± 0.10	2.41 ± 2.09	1.48 ± 2.01	8.51 ± 0.07

**Table 3:** Hardness of the composites.

Composite (Phr)	Shore OO Harness	
	NR/UJF	NR/TJF
0	81.8 ± 3	
1	78.4 ± 1	79.3 ± 2
2	79.9 ± 1	79.8 ± 2
3	78.5 ± 2	79.8 ± 2
4	81.1 ± 2	79.2 ± 3
5	79.8 ± 2	79.9 ± 3
10	78.1 ± 2	79.6 ± 3

**Table 4:** Results of the abrasion resistance tests on the NR/UJF and NR/TJF composites.

Amount (Phr)	Volume Loss (mm^3^)		Abrasion Resistance (%)	
	NR/UJF	NR/TJF	NR/UJF	NR/TJF
0	180.4 ± 3		87.2 ± 3	
1	258.5 ± 2	289.3 ± 2	60.3 ± 1	43.3 ± 3
2	289.3 ± 2	377.1 ± 2	53.9 ± 1	64.4 ± 3
3	377.1 ± 2	210.2 ± 2	41.4 ± 1	65.8 ± 3
4	210.2 ± 2	218.3 ± 2	74.4 ± 2	80.1 ± 2
5	218.3 ± 2	269.4 ± 2	71.5 ± 2	71.6 ± 2
10	269.4 ± 2	228.1 ± 3	58.0 ± 2	68.3 ± 2

## Data Availability

The data are contained in the article.
